# Initial validation of an intelligent video surveillance system for automatic detection of dairy cattle lameness

**DOI:** 10.3389/fvets.2023.1111057

**Published:** 2023-06-13

**Authors:** Alkiviadis Anagnostopoulos, Bethany E. Griffiths, Nektarios Siachos, Joseph Neary, Robert F. Smith, Georgios Oikonomou

**Affiliations:** Department of Livestock and One Health, Institute of Infection, Veterinary and Ecological Sciences, University of Liverpool, Leahurst Campus, Chester, United Kingdom

**Keywords:** cattle lameness, automated system, foot lesions, mobility scoring, artificial intelligence

## Abstract

**Introduction:**

Lameness is a major welfare challenge facing the dairy industry worldwide. Monitoring herd lameness prevalence, and early detection and therapeutic intervention are important aspects of lameness control in dairy herds. The objective of this study was to evaluate the performance of a commercially available video surveillance system for automatic detection of dairy cattle lameness (CattleEye Ltd).

**Methods:**

This was achieved by first measuring mobility score agreement between CattleEye and two veterinarians (Assessor 1 and Assessor 2), and second, by investigating the ability of the CattleEye system to detect cows with potentially painful foot lesions. We analysed 6,040 mobility scores collected from three dairy farms. Inter-rate agreement was estimated by calculating percentage agreement (PA), Cohen’s kappa (*κ*) and Gwet’s agreement coefficient (AC). Data regarding the presence of foot lesions were also available for a subset of this dataset. The ability of the system to predict the presence of potentially painful foot lesions was tested against that of Assessor 1 by calculating measures of accuracy, using lesion records during the foot trimming sessions as reference.

**Results:**

In general, inter-rater agreement between CattleEye and either human assessor was strong and similar to that between the human assessors, with PA and AC being consistently above 80% and 0.80, respectively. Kappa agreement between CattleEye and the human scorers was in line with previous studies (investigating agreement between human assessors) and within the fair to moderate agreement range. The system was more sensitive than Assessor 1 in identifying cows with potentially painful lesions, with 0.52 sensitivity and 0.81 specificity compared to the Assessor’s 0.29 and 0.89 respectively.

**Discussion:**

This pilot study showed that the CattleEye system achieved scores comparable to that of two experienced veterinarians and was more sensitive than a trained veterinarian in detecting painful foot lesions.

## 1. Introduction

Lameness poses a major challenge to the dairy industry worldwide and has a well-documented negative impact on dairy cattle milk production, fertility and longevity (1–3). Apart from the financial implications of lameness its impact on animal welfare cannot be understated (4, 5). Early lameness detection has been shown to be an important aspect of lameness management in dairy herds (6) and yet for the most part relies on visual mobility/locomotion scoring by farm staff or trained scorers. This process, albeit useful, is time consuming, labour intensive and subjective even when agreement within the same experienced assessor is examined (7). Furthermore, farmers have been shown to significantly underestimate lameness prevalence in their herd (8). An automated system that could reliably identify lame cows would not only have the advantage of being objective and consistent but could also provide daily information about the lameness status of the herd.

CattleEye Ltd. (Belfast, United Kingdom) has recently developed and commercialised a system for automatic lameness detection. This system is the first to utilize inexpensive 2D surveillance cameras placed above the passageway exiting the milking parlour. Footage of cows exiting the milking parlour is sent directly to company servers where it is stored and processed. The footage analysis requires a minimum of 40 frames recorded over 2 s (20 fps setting). Initially, an object-tracking algorithm is used to identify the outline of the body and track it across frames. Based on information gathered during the enrolment of the herd, the algorithm identifies the individual animal (based on coat pattern and head shape) and assigns its identification number to the recording. Specific reference points are marked and their coordinates across frames are recorded on a matrix. This information is then processed by the convolutional neural network and the average pooling output is used during the linear activation stage to produce a mobility score. The final result of the analysis is a floating-point number between 0 and 100, indicating the degree of lameness in relation to changes observed between reference points in each frame and between frames. For example, a score of 0 indicates good mobility whilst a score of 100 would indicate a very poor level of mobility and therefore a very high likelihood of lameness.

The objective of this pilot study was to evaluate the performance of this video surveillance system for automatic detection of dairy cattle lameness. Our aim was to investigate the agreement between the mobility scores provided by the CattleEye system and the mobility scores recorded by two experienced veterinarians. Additionally, we examined the system’s ability to detect cows with potentially painful foot lesions.

## 2. Materials and methods

### 2.1. Farms’ characteristics and animals

From November 2020 to February 2021 three commercial dairy farms in Northwest England and North Wales participated in this validation study. All farms milked Holsteins cows that were housed during the study period and were already equipped with the CattleEye mobility scoring system. Farm 1 housed all year round a milking herd of *ca.* 180 cows. Farm 2 consisted of a milking herd of *ca.* 340 cows. Freshly calved and early lactation cows were housed year-round while late lactation cows were grazed during spring and summer. Farm 3 housed a milking herd of *ca.* 750 cows all year round. Farm staff were responsible for foot trimming in Farm 1. Farms 2 and 3 used the same professional foot trimmer who was performing routine and therapeutic foot trimming on each farm fortnightly.

### 2.2. CattleEye mobility scoring system

The CattleEye scoring system produces scores on a scale from 0 to 100, with each 25-increment representing one grade on the UK Agricultural and Horticultural Development Board (AHDB) mobility scoring system (9). More specifically, cows with a score <25 were graded as 0, those with a score ≥25 and <50 were graded as 1, those with a score ≥50 and <75 were graded as 2, and those with a score ≥75 were graded as 3. The four-grade mobility score variable that was produced by this transformation will be hereinafter referred to as the CattleEye mobility score (CE_MS).

### 2.3. Mobility scoring records

During the study, all three farms were visited approximately once a week by an experienced scorer (Assessor 1, AA) who was a veterinarian trained by an expert in dairy cattle lameness and had been working exclusively on cattle lameness research for a three-year period prior to the commencement of this study. During each visit, the entire milking herd was scored by Assessor 1 using the AHDB 0–3 four-grade scale scoring method (9). Reports containing CE_MS (weekly average for each cow) were also made available to the corresponding author (GO) of this study. Importantly, Assessor 1 did not have access to the CattleEye data and CattleEye Ltd. did not have access to the Assessor’s scores. At the end of the validation period, the Assessor’s records for each visit were merged with the corresponding CE_MS (for the week prior to the assessor’s visit) using the cow identification numbers. Records from all visits were then combined to create Dataset A.

A second experienced assessor (Assessor 2, BG), a veterinarian accredited by the Register of Mobility Scorers (Register of Mobility Scorers Limited, Wimborne, United Kingdom) and trained by the same expert as Assessor 1, recorded mobility scores once on Farms 2 and 3. Assessor 2 evaluated cows on Farm 2 simultaneously with Assessor 1 and within 48 h from one of Assessor 1 scoring sessions on Farm 3. Assessors had no knowledge of each other’s scores prior to or during the visit. Dataset B contained the individual mobility scores recorded by Assessor 1 and Assessor 2, and the corresponding CE_MS.

### 2.4. Foot lesion records

Assessor 1 was present during professional foot trimming sessions on Farm 2 and Farm 3 and soon after a mobility scoring session in order to consistently record presence of foot lesions. These included both routine and therapeutic trims and by the end of the study foot lesion data from 84 cows were recorded according to the ICAR claw health atlas (10). Lesions were graded for severity on a scale from 0 to 3 as described in Supplementary Table S1. Assessor 1 had no prior knowledge regarding which cows were sorted for routine trimming and which for therapeutic foot trimming. Lesion records were merged with Assessor 1 mobility scores and CE_MS obtained at the closest date prior to the foot trimming session to create Dataset C. An overall binary lesion score was generated (Lesion_BIN) with 1 representing cows that were found with at least one potentially painful lesion and 0 cows with milder or no lesions. Lesions described as potentially painful for the purposes of this classification were: sole ulcer lesions of grade >0, white line lesions of grade 3, toe ulcer lesions of grade >0, interdigital hyperplasia lesions of grade >1 and digital dermatitis lesions of grade 3.

### 2.5. Statistical analysis

Data were handled and analysed using R 3.6.

In all datasets, the four grade (0–3) mobility scores recorded by Assessor 1 (A1_MS), Assessor 2 (A2_MS) and CE_MS were also transformed into binary variables (0,1/2,3; non-lame/lame), namely A1_BIN, A2_BIN and CE_BIN, respectively.

Agreement between A1_MS and CE_MS (Dataset A) and between A1_MS, A2_MS and CE_MS (Dataset B; all pairwise combinations) was estimated by calculating the weighted Cohen’s kappa (w*κ*) and the weighted Gwet’s coefficient (AC_2_) using quadratic weights.

Agreement between A1_BIN and CE_BIN (Dataset A) and between A1_BIN, A2_BIN and CE_BIN (Dataset B; all pairwise combinations) was estimated by calculating the percentage of agreement (PA), unweighted Cohen’s kappa (*κ*), and the unweighted Gwet’s coefficient (AC_1_). Finally on dataset B, confusion matrixes (11) were created to calculate measures of accuracy (sensitivity (SE) and specificity (SP)) of A2_BIN and CE_BIN in predicting A1_BIN scores.

Interpretation of each agreement coefficient was according to the Landis and Koch (12) recommendations: values 0.00–0.20: slight agreement; values 0.21–0.40: fair agreement; values 0.41–0.60: moderate agreement; values 0.61–0.80: substantial agreement; values 0.81–1.00: almost perfect agreement. The benchmark of acceptable reliability used in this study was ≥0.60 for *κ*, w*κ*, AC_1_, and AC_2_ (13, 14).

Using dataset C, confusion matrixes were created to calculate measures of accuracy (SE; SP; positive predictive value (PPV), and negative predictive value (NPV)) of A1_BIN and CE_BIN in predicting the presence of potentially painful lesions, using Lesion_BIN as reference.

## 3. Results

**Figure 1:**
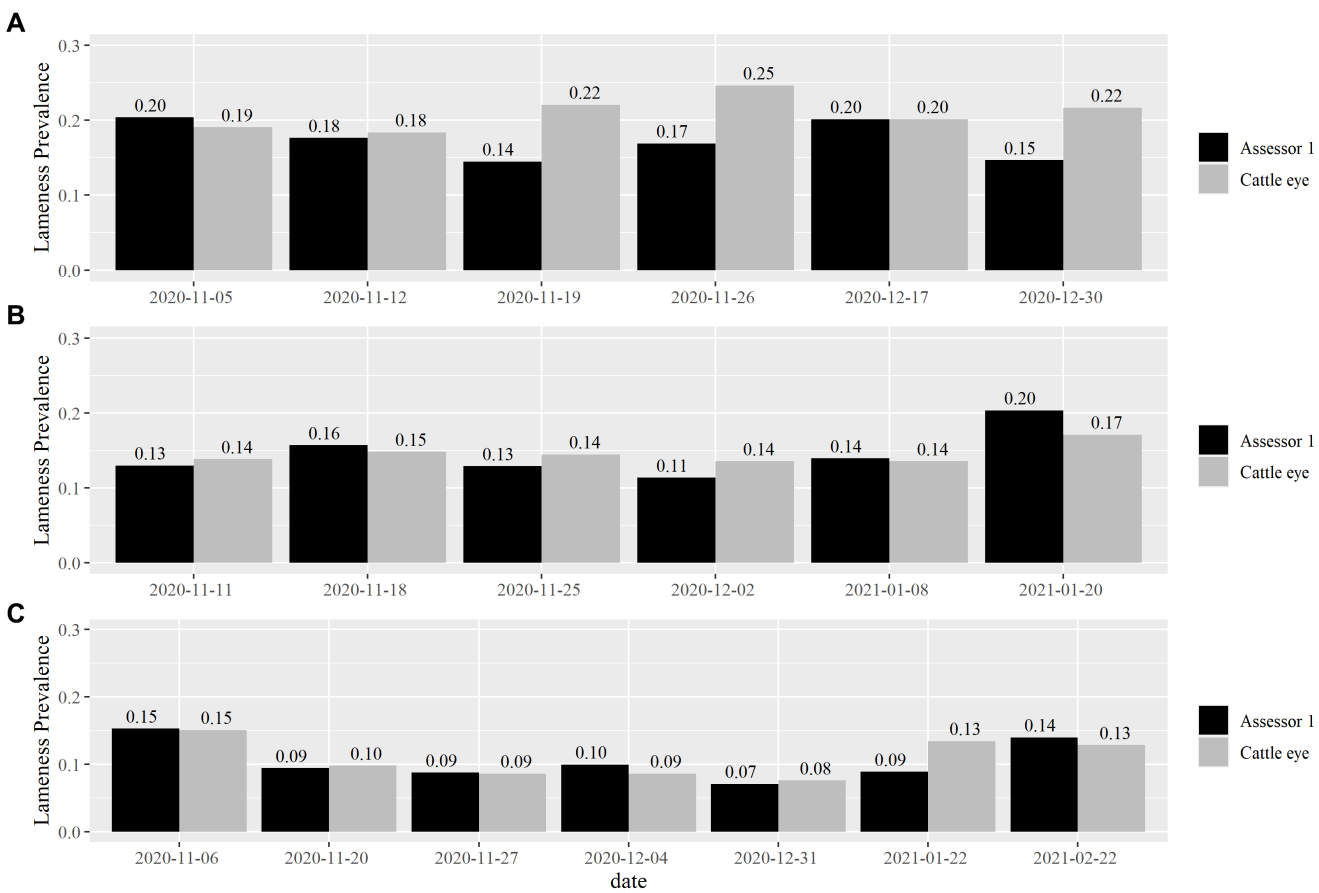
Lameness prevalence as recorded by Assessor 1 and the Cattle-eye system for each farm visit. Cows with mobility scores 2 and 3 were scored as lame. Data from each farm is presented separately (**A** for Farm 1, **B** for Farm 2 and **C** for Farm 3).

The total number of records for each mobility score grade and farm according to all scorers for Dataset A and Dataset B are summarized in Table 1. Lameness prevalence for each farm and visit as recorded by Assessor 1 and by CattleEye is presented in Figure 1. Herd lameness prevalence ranged from 7 to 20% and from 8 to 25% between farm visits, according to Assessor 1 and CattleEye, respectively.

**Table 1:** Summary of four grade (0–3) mobility scores recorded by Assessor 1 and CattleEye for farms 1, 2, and 3 (Dataset A) and scores collected by both assessors and CattleEye for farms 2 and 3 (Dataset B).

Farm	1	2	3
Dataset A			
Observations	*n* = 857	*n* = 1,387	*n* = 3,796
Assessor 1 Mobility Score (A1_MS)			
0	141 (16%)	223 (16%)	1,007 (27%)
1	567 (66%)	971 (70%)	2,399 (63%)
2	131 (15%)	171 (12%)	342 (9.0%)
3	18 (2.1%)	22 (1.6%)	48 (1.3%)
CattleEye Mobility Scores (CE_MS)			
0	90 (11%)	285 (21%)	1,499 (39%)
1	573 (67%)	885 (64%)	1,846 (49%)
2	194 (23%)	215 (16%)	441 (12%)
3	0 (0%)	2 (0.1%)	10 (0.3%)
Dataset B			
Assessor 1 Mobility Score (A1_MS)			
0		27 (22%)	214 (37%)
1		73 (58%)	312 (54%)
2		25 (20%)	43 (7.5%)
3		0 (0%)	8 (1.4%)
CattleEye Mobility Scores (CE_MS)			
0		45 (17%)	230 (36%)
1		182 (67%)	310 (49%)
2		43 (16%)	89 (14%)
3		1 (0.4%)	3 (0.5%)
Assessor 2 Mobility Score (A2_MS)			
0		36 (29%)	332 (56%)
1		71 (57%)	190 (32%)
2		17 (14%)	61 (10%)
3		1 (0.8%)	5 (0.9%)

### 3.1. Inter-rater agreement

Dataset A consisted of a total of 6,040 paired mobility scoring records (Farm 1: 857; Farm 2: 1,387, and Farm 3: 3,796). Agreement between Assessor 1 and CattleEye mobility scores and binary scores is summarized in Table 2.

**Table 2:** Inter-rater agreement of mobility score between Assessor 1 and the CattleEye system, estimated with weighted Cohen’s kappa (w*κ*) and weighted Gwet’s agreement coefficient type 2 (AC_2_) for the 4-grade scoring (0–3) and with percentage agreement (PA), unweighted Cohen’s kappa (*κ*) and Gwet’s agreement coefficient type 1 (AC_1_) for the binary transformed 2-grade scoring (0,1/2,3).

Farm	*n*	PA	*κ*/w*κ*	AC_1_/AC_2_
1	857			
0–3			0.405	0.868
0,1/2,3		82.6%	0.441	0.747
2	1,387			
0–3			0.347	0.862
0,1/2,3		84.1%	0.342	0.789
3	3,796			
0–3			0.369	0.820
0,1/2,3		88.9%	0.411	0.863
All	6,040			
0–3			0.386	0.835
0,1/2,3		86.9%	0.404	0.832

*n*, number of observations.

Cohen’s w*κ* for agreement between A1_MS and CE_MS was >0.40 only on Farm 1. On the other hand, AC_2_ was consistently >0.80 with an overall value of 0.835, indicating almost perfect agreement.

Percentage agreement between A1_BIN and CE_BIN was 87%; ranging from 82.6 to 88.9% between different farms. The overall agreement was fair using the Cohen’s *κ* coefficient (*κ* = 0.404), while AC_1_ was within the range of almost perfect agreement (AC_1_ = 0.832).

Dataset B included observations from a total of 903 cows (Farm 2: 271, and Farm 3: 632). Agreement between A1_MS, A2_MS and CE_MS is shown in Table 3. Regarding Cohen’s w*κ*, moderate agreement (w*κ* > 0.40) was only achieved between A1_MS and A2_MS. According to AC_2_, agreement bellow the almost perfect range was only observed between A2_MS and CE_MS where AC_2_ was 0.79 and 0.78 for Farms 2 and 3, respectively.

**Table 3:** Inter-rater agreement of mobility score combinations between Assessor 1, Assessor 2, and the CattleEye system in Farms 2 and 3, estimated with weighted Cohen’s kappa (w*κ*) and weighted Gwet’s agreement coefficient type 2 (AC_2_) for the 4-grade mobility score (0–3) and with percentage agreement (PA), unweighted Cohen’s kappa (*κ*) and Gwet’s agreement coefficient type 1 (AC_1_) for the binary transformed 2-grade mobility score (0,1/2,3).

Farm	*n*	PA	*κ*/w*κ*	AC_1_/AC_2_	PA	*κ*/w*κ*	AC_1_/AC_2_	PA	*κ*/w*κ*	AC_1_/AC_2_
		Assessor 1 vs. Assessor 2			Assessor 1 vs. CattleEye			Assessor 2 vs. CattleEye		
2	271									
0–3			0.347	0.827		0.258	0.810		0.210	0.786
0,1/2,3		80.0%	0.382	0.720	77.6%	0.255	0.679	81.6%	0.302	0.750
3	632									
0–3			0.407	0.808		0.386	0.808		0.379	0.776
0,1/2,3		90.1%	0.442	0.879	88.0%	0.401	0.850	85.0%	0.325	0.806
All	903									
0–3			0.418	0.808		0.377	0.806		0.366	0.772
0,1/2,3		88.2%	0.408	0.853	86.2%	0.368	0.823	86.2%	0.321	0.797

*n*, number of observations.

Percentage agreement between A1_BIN and CE_BIN and between A2_BIN and CE_BIN were the same (86.2%); a similar PA was also produced for the agreement between A1_BIN and A2_BIN (88.2%). According to Cohen’s *κ* fair agreement was observed between all possible pairs across farms except for the pairs A1_BIN/A2_BIN and A1_BIN/CE_BIN where moderate agreement was achieved for Farm 3 (0.44 and 0.40 respectively). The overall AC_1_ was ≥0.80 for all possible pairs, indicating almost perfect agreement.

According to the confusion matrixes produced, both A2_BIN and CE_BIN had almost the same ability to predict A1_BIN scores achieving combinations of 51% SE, 92% SP and 51% SE, 90% SP, respectively.

### 3.2. Detection of painful foot lesions

A summary of the lesions recorded throughout the study for Dataset C is presented on Table 4. Using Lesion_BIN as reference and CE_BIN as a predictor, the confusion matrix produced a combination of 52% SE and 81% SP in predicting the presence of potentially painful foot lesions with an accuracy of 73.81%. Positive and negative predictive values were 0.48 and 0.84, respectively. Using A1_BIN as a predictor, the confusion matrix produced a combination of 29% SE and 89% SP with an accuracy of 73.81%. Positive and negative predictive values were 0.46 and 0.79, respectively.

**Table 4:** Total number and percentage of foot lesions and severity (Dataset C).

	*N*	%
Farm		
2	42	50%
3	42	50%
Severity* SH	*n*	
0	21	25%
1	21	25%
2	29	35%
3	13	15%
SU		
0	72	86%
1	6	7.1%
2	5	6%
3	1	1.2%
WL		
0	48	57%
1	11	13%
2	20	24%
3	5	6%
TU		
0	83	99%
3	1	1.2%
IH		
0	78	93%
1	2	2.4%
2	4	4.8%
DD		
0	68	81%
1	9	11%
2	3	3.6%
3	4	4.8%

*N*, number of cows examined; *n*, number of lesions observed; SH, sole haemorrhage; SU, sole ulcer; WL, white line lesion; TU, toe ulcer; IH, interdigital dermatitis; DD, digital dermatitis. ^*^: severity of the lesions recorded on a 0–3 scale with 0 representing absence and 3 representing the most sever stages of the lesion.

## 4. Discussion

We have shown here that the CattleEye automatic lameness detection system performs similarly to two well trained veterinarians by calculating 3 different measures of inter-rater agreement (PA, Cohen’s *κ* and Gwet’s AC) for both the 4-grade (0–3) and the binary converted 2-grade (0,1/2,3) mobility scores. Overall, PAs were >80% and AC were constantly above the benchmarks of accepted reliability, while *κ* coefficients were low, indicating only fair to moderate agreement.

Kappa agreement between Assessor 1, Assessor 2, and CattleEye fell within the range described by Thomsen et al. (15) when inter-observer agreement was investigated (*κ* values ranged from 0.24 to 0.68). Linardopoulou et al. (16) recently reported very low to moderate *κ* coefficients (0.004 to 0.565) between multiple human assessors; results were affected by scoring method used and the farm visit. Higher *κ* values for inter-observer agreement have been reported by others (7, 17, 18), but those studies involved scoring cows using a relatively small number of video recordings trying to equally represent all mobility grades. Our study was conducted under commercial farm conditions and scorers had to record cow ID and evaluate mobility scores for 100 of cows exiting the milking parlour often having just a few seconds for each animal; this is how mobility scoring is performed in practice.

The discrepancy between AC and *κ* could be due to a statistical phenomenon called the kappa paradox. This phenomenon is defined by low *κ* values in the presence of high percent agreement, under the influence of raters’ classification probabilities and low prevalence of the tested trait (19). Paradoxical situations, when using *κ* to test inter-observer agreement, have been reported across various medical fields (20, 21). As a result, the use of Gwet’s AC (22) is becoming popular as it is considered a more stable coefficient, especially in low prevalence scenarios. To the best of the authors’ knowledge, there are no published studies estimating inter-rater agreement in mobility scoring using Gwet’s coefficients to compare to ours. Using AC_2_, agreement between the two human assessors was almost perfect in Dataset B. Better results were obtained using AC_1_ for the binary scores for Farm 3. Agreement of CattleEye with either human assessor was about the same and very similar to that between the two human assessors, and always above the benchmark of accepted reliability.

The impact of hoof pathologies on cows’ gait is a proven concept (23) that has been recorded using kinematic techniques. Song et al. (24) described one of the first fully automated methods of recording trackway and gait characteristics. Utilizing kinematic techniques based on leg swing, Zhao et al. (25) developed an algorithm that achieved 90.18% accuracy on a tenfold cross validation using a total number of 621 video recordings of 98 cows. Both Viazzi et al. (26) and Poursaberi et al. (27) utilized the Body Movement Pattern that emphasizes on back curvature. They later automated this method and when tested on 1,200 video recordings of cows only 88 where misclassified by the algorithm (28). For the most part these systems involve video recordings of individual cows using as gold standard the mobility score provided by a scorer after evaluating the recording and not comparing human scorers against an automatic system in real time on commercial farm settings.

In our study, binary converted CattleEye scores achieved the same accuracy as Assessor 1 (when lesion detection was evaluated), being actually more sensitive in predicting the presence of potentially painful lesions. However, the SE produced by CattleEye was still relatively low, allowing for a high proportion of cows gone undetected (false negatives). On the other hand, SP was high, allowing only for a small percentage of false positives with the human assessor performing slightly better. Both human assessor and CattleEye produced low PPV and high NPV, with CattleEye performing slightly better. This suggests that, within the herd lameness prevalence observed in this study, a cow being assigned a “negative” score (0,1: non-lame) either by a human assessor or the automated detection system has high odds of actually not baring potentially painful foot lesions.

The ability of CattleEye to outperform the human scorer in the detection of severe lesions when sensitivity is concerned might be due to the innate advantages of automatic systems and the frequency of scoring. Human assessor scoring is prone to errors and misclassifications due to various practical reasons, besides subjectivity. The human scorer only had a few seconds for each individual cow once a week. Circumstances when multiple cows exit the parlour at the same time disturbing the flow and scoring process are quite common in most farms. Difficult weather conditions and fatigue due to long hours of repeatedly scoring large herds may also add to the chance of human error. In contrast, an automated system is less prone to such errors. The system is able to assess each cow after each milking, every day, potentially reaching 14 to 21 scores for each individual cow per week. This ability guarantees that momentary disturbances to cow flow would not affect the average weekly score. Additionally, normal idiosyncrasies in an animal’s gait are recognised by the algorithm. In other words, a slight change in movement pattern that would not justify a classification of a cow as lame by a human assessor might be highly irregular for a certain animal based on saved footage history and thus increasing the CE_MS algorithm above the lameness threshold.

Our study has several limitations. The intra-rater agreement of each human assessor and of the automated system was not considered. Therefore, we cannot acknowledge whether the lack of precision of each assessor influenced the observed inter-rater agreement. Ideally, multiple assessors of varying experience could have recorded mobility scores on all farms involved in this study. That way the deviation of each Assessor and the CattleEye system from the mean could have been calculated. Additionally, more lesions could have been recorded close to a mobility scoring visit to use as the gold standard of lameness detection. This should be the scope of future studies.

Based on the ability of CattleEye and Assessor 2 mobility scores to predict the binary scores recorded by Assessor 1, the agreement between all possible pairs, and the literature describing mobility score agreement between human assessors, it is not unreasonable to describe the system’s performance as equivalent to that of a trained scorer. Granted there was slightly better agreement between the two human assessors but that is to be expected since they had the same training and working environment for more than 2 years. Future investigations should consider the addition of external professional mobility score assessors of various experience and background to put those differences in the calculated agreement into perspective. The system was more sensitive in identifying lameness causing lesions compared to Assessor 1. This further justifies the use of the CattleEye system not just as a herd lameness prevalence monitoring system, but rather as an early lameness detection aid for individual cows. Training CattleEye algorithms using large datasets containing foot lesion information could further improve its ability for early detection of foot pathology.

## 5. Conclusion

We showed that the CattleEye system is producing mobility scores comparable to those of two experienced scorers with similar training. When it came to lesion detection the system was more sensitive than the human scorer and achieved the same accuracy. Implementing a system that can produce reliable mobility scores for each animal multiple times per week (or even daily) regardless of herd size, could prove an invaluable tool in lameness management. Automatic lameness detection is not prone to subjectivity and fatigue in contrast to human scorers and the system’s ability to detect lesions can aid in early treatment minimising production loss and improving animal welfare.

## Data availability statement

The raw data supporting the conclusions of this article will be made available by the authors, without undue reservation.

## Ethics statement

The animal study was reviewed and approved by University of Liverpool Veterinary Research Ethics Committee. Written informed consent was obtained from the owners for the participation of their animals in this study.

## Author contributions

Data collection was undertaken by AA and BG. Statistical analysis was undertaken by AA and assisted by GO and NS. The manuscript draft was written by AA with significant contributions from GO and NS. GO (corresponding author) conceived and designed the study with significant contributions from RS and JN. All authors contributed to the article and approved the submitted version.

## Funding

CattleEye Ltd. provided funding and approved the final version of this manuscript. The funder participated in the discussions leading to the study design but had no involvement in data collection and analysis.

## Conflict of interest

The authors declare that the research was conducted in the absence of any commercial or financial relationships that could be construed as a potential conflict of interest.

## Publisher’s note

All claims expressed in this article are solely those of the authors and do not necessarily represent those of their affiliated organizations, or those of the publisher, the editors and the reviewers. Any product that may be evaluated in this article, or claim that may be made by its manufacturer, is not guaranteed or endorsed by the publisher.

## References

[1] Booth CJ Warnick LD Gröhn YT Maizon DO Guard CL Janssen D. Effect of lameness on culling in dairy cows. J Dairy Sci. (2004) 87:4115–22. doi: 10.3168/jds.S0022-0302(04)73554-7 15545373

[2] Melendez P Bartolome J Archbald LF Donovan A. The association between lameness, ovarian cysts and fertility in lactating dairy cows. Theriogenology. (2003) 59:927–37. doi: 10.1016/S0093-691X(02)01152-4, PMID: 12517394

[3] Puerto MA Shepley E Cue RI Warner D Dubuc J Vasseur E. The hidden cost of disease: II. Impact of the first incidence of lameness on production and economic indicators of primiparous dairy cows. J Dairy Sci. (2021) 104:7944–55. doi: 10.3168/jds.2020-19585, PMID: 33865579

[4] Bruijnis MRN Beerda B Hogeveen H Stassen EN. Assessing the welfare impact of foot disorders in dairy cattle by a modeling approach. Animal. (2012) 6:962–70. doi: 10.1017/S1751731111002606, PMID: 22558967

[5] Whay HR Waterman AE Webster AJF. Associations between locomotion, claw lesions and nociceptive threshold in dairy heifers during the peri-partum period. Vet J. (1997) 154:155–61. doi: 10.1016/S1090-0233(97)80053-6, PMID: 9308402

[6] Groenevelt M Main DCJ Tisdall D Knowles TG Bell NJ. Measuring the response to therapeutic foot trimming in dairy cows with fortnightly lameness scoring. Vet J. (2014) 201:283–8. doi: 10.1016/j.tvjl.2014.05.017, PMID: 24881511

[7] Garcia E König K Allesen-Holm BH Klaas IC Amigo JM Bro R. Experienced and inexperienced observers achieved relatively high within-observer agreement on video mobility scoring of dairy cows. J Dairy Sci. (2015) 98:4560–71. doi: 10.3168/jds.2014-9266, PMID: 25935241

[8] Beggs DS Jongman EC Hemsworth PE Fisher AD. Lame cows on Australian dairy farms: a comparison of farmer-identified lameness and formal lameness scoring, and the position of lame cows within the milking order. J Dairy Sci. (2019) 102:1522–9. doi: 10.3168/jds.2018-14847, PMID: 30594372

[9] Whay HR Main DC Green LE Webster AJ. Assessment of the welfare of dairy cattle using animal-based measurements: direct observations and investigation of farm records. Vet Rec. (2003) 153:197–202. doi: 10.1136/vr.153.7.197, PMID: 12956296

[10] Egger-Danner C. Nielsen P. Fiedler A. Müller K. Fjeldaas T. Döpfer D., (2014). ICAR claw health atlas.

[11] Max A. Wing J. Weston S. Williams A. Keefer C. Engelhardt A., (2021). Package ‘Caret’ R topics documented

[12] Landis JR Koch GG. The measurement of observer agreement for categorical data. Biometrics. (1977) 33:159–74. doi: 10.2307/2529310 843571

[13] Berman J Francoz D Abdallah A Dufour S Buczinski S. Evaluation of inter-rater agreement of the clinical signs used to diagnose bovine respiratory disease in individually housed veal calves. J Dairy Sci. (2021) 104:12053–65. doi: 10.3168/jds.2021-20503, PMID: 34454767

[14] Schlageter-Tello A Bokkers EAM Groot Koerkamp PWG Van Hertem T Viazzi S Romanini CEB. Effect of merging levels of locomotion scores for dairy cows on intra- and interrater reliability and agreement. J Dairy Sci. (2014) 97:5533–42. doi: 10.3168/jds.2014-8129, PMID: 24996266

[15] Thomsen PT Munksgaard L Togersen FA. Evaluation of a lameness scoring system for dairy cows. J Dairy Sci. (2008) 91:119–26. doi: 10.3168/jds.2007-0496 18096932

[16] Linardopoulou K. Viora L. Fioranelli F. Kernec J. Abbasi Q.. (2022). Time-series observations of cattle mobility: accurate label assignment from multiple assessors, and association with lesions detected in the feet. Proceedings of the 31st world Buiatrics congress, p 297.

[17] Dahl-Pedersen K Foldager L Herskin MS Houe H Thomsen PT. Lameness scoring and assessment of fitness for transport in dairy cows: agreement among and between farmers, veterinarians and livestock drivers. Res Vet Sci. (2018) 119:162–6. doi: 10.1016/j.rvsc.2018.06.017, PMID: 29940460

[18] Gardenier J Underwood J Weary DM Clark CEF. Pairwise comparison locomotion scoring for dairy cattle. J Dairy Sci. (2021) 104:6185–93. doi: 10.3168/jds.2020-19356, PMID: 33663829

[19] Byrt T Bishop J Carlin JB. Bias, prevalence, and kappa. J Clin Epidemiol. (1993) 46:423–9. doi: 10.1016/0895-4356(93)90018-V 8501467

[20] Cibulka MT Strube MJ. The conundrum of kappa and why some musculoskeletal tests appear unreliable despite high agreement: a comparison of cohen kappa and gwet ac to assess observer agreement when using nominal and ordinal data. Phys Ther. (2021) 101:1–5. doi: 10.1093/ptj/pzab150, PMID: 34132806

[21] Wongpakaran N Wongpakaran T Wedding D Gwet KL. A comparison of Cohen’s kappa and Gwet’s AC1 when calculating inter-rater reliability coefficients: a study conducted with personality disorder samples. BMC Med Res Methodol. (2013) 13:1–7. doi: 10.1186/1471-2288-13-61, PMID: 23627889 PMC3643869

[22] Gwet KL. Computing inter-rater reliability and its variance in the presence of high agreement. Br J Math Stat Psychol. (2008) 61:29–48. doi: 10.1348/000711006X126600, PMID: 18482474

[23] Flower FC Sanderson DJ Weary DM. Hoof pathologies influence kinematic measures of dairy cow gait. J Dairy Sci. (2005) 88:3166–73. doi: 10.3168/jds.S0022-0302(05)73000-9, PMID: 16107407

[24] Song X Leroy T Vranken E Maertens W Sonck B Berckmans D. Automatic detection of lameness in dairy cattle-vision-based trackway analysis in cow’s locomotion. Comput Electron Agric. (2008) 64:39–44. doi: 10.1016/j.compag.2008.05.016

[25] Zhao K Bewley JM He D Jin X. Automatic lameness detection in dairy cattle based on leg swing analysis with an image processing technique. Comput Electron Agric. (2018) 148:226–36. doi: 10.1016/j.compag.2018.03.014

[26] Viazzi S Bahr C Schlageter-Tello A Van Hertem T Romanini CEB Pluk A. Analysis of individual classification of lameness using automatic measurement of back posture in dairy cattle. J Dairy Sci. (2013) 96:257–66. doi: 10.3168/jds.2012-5806, PMID: 23164234

[27] Poursaberi A Bahr C Pluk A Van Nuffel A Berckmans D. Real-time automatic lameness detection based on back posture extraction in dairy cattle: shape analysis of cow with image processing techniques. Comput Electron Agric. (2010) 74:110–9. doi: 10.1016/j.compag.2010.07.004

[28] Poursaberi A Bahr C Pluk A Berckmans D Veermäe I Kokin E. Online lameness detection in dairy cattle using body movement pattern (BMP). Int Conf Intell Syst Des Appl ISDA. (2011):732–6. doi: 10.1109/ISDA.2011.6121743

