# Supplementary material for: Initial validation of an intelligent video surveillance system for automatic detection of dairy cattle lameness
Source: Front Vet Sci. 2023 Jun 13;10:1111057. doi: 10.3389/fvets.2023.1111057 (PMC10299827; doi:10.3389/fvets.2023.1111057)
Supplement: Supplementary file 1 [file Table_1.docx]

**Table S1. Case and severity definition for foot lesion scoring**

| Grade | 0 | 1 | 2 | 3 |
| --- | --- | --- | --- | --- |
| Sole Haemorrhage (SH) | Absence of lesion | Lesion smaller than 2cm light pink in colour | Lesion wider than 2cm in diameter of light pink coloration or dark red coloured lesion smaller than 2 cm | Dark red coloured lesion wider than 2cm or blue coloured lesion of any size |
| Sole Ulcer (SU) | Absence of lesion | Small ulceration with less than 2cm diameter | Ulceration 2cm in diameter or wider and/or soft tissue less than 1,5cm in size protruding through the horn | Soft tissue larger than 1.5cm is exposed protruding through the horn and/or secondary infection and necrosis is present and/or fistulas or abscess present |
| White Line (WL) | Absence of lesion | mild discoloration, haemorrhage or separation is observed on the white line that is not visible after trimming | Deep separation or discoloration of the white line. The discoloration is still present after trimming with and soft tissue might be exposed if excision is attempted | Fissure, with the corium involved and/or purulent exudate or necrosis. Fistulas might be found connecting the lesion to the coronary band or underrunning the sole |
| Toe Ulcer (TU) | Absence of lesion | Small ulceration with less than 2cm diameter | Ulceration 2cm in diameter or wider and/or soft tissue less than 1,5cm in size protruding through the horn | Soft tissue larger than 1.5cm is exposed protruding through the horn and/or secondary infection and toe necrosis is present and/or abscess present |
| Interdigital Hyperplasia (IH) | Absence of lesion | Fibrous tissue flap on the interdigital skin that does not increase the distance between claws | The claw distance is increased due to the growth of the interdigital fibrous tissue. | The interdigital growth increases the distance between claws and shows signs of inflammation and/or traumatic bleeding |
| Digital Dermatitis (DD) | Absence of lesion | M4 and M3 stages of digital dermatitis | M4.1 and M1 stages of digital dermatitis | M2 stage of digital dermatitis |
